# Œsophageal Obstruction.—II

**Published:** 1908-08-08

**Authors:** 


### Surgery.

### ŒSOPHAGEAL OBSTRUCTION—II.

### FOREIGN BODIES IN THE ŒSOPHAGUS.

It has already been said that it is quite possible for the ingestion of a foreign body to give rise to symptoms of œsophageal obstruction. In such a case it is not the obstruction to the passage of food, but the actual presence of the foreign body in the œsophagus which causes the trouble. But, in any case, the presence of a foreign body impacted in the œsophagus calls urgently for immediate removal by appropriate surgical or other measures.

First the patient should be $x$-rayed, if the swallowed body consists altogether or in part, as it usually does, of metal or other substances which give an opaque shadow with the rays. The body is usually found to be impacted at one of the three situations which have already been mentioned as being the favourite sites of carcinoma—namely, (i.) opposite the cricoid; (ii.) where the œsophagus is crossed by the left bronchus; and (iii.) at the cardiac orifice of the stomach, these being the narrowest points in the structure.

The body having been located, the various instruments which have been designed for extracting it *viâ* the mouth, may be tried in turn. The œsophageal probang is, in the writer's experience, quite useless, and he cannot recall a single case in which it has been successful, except in the case of a fishbone fixed in the highest part of the œsophagus. The curved œsophageal forceps and the coin-catcher are both more serviceable instruments. But if they are used, it must be remembered that only gentle traction must be applied, for the œsophageal wall is damaged with extraordinary ease, and tearing it may lead to serious or even fatal complications. Special care must be taken when the body swallowed is a toothplate, for these are nearly always armed with sharp hooks or prongs, and a moment's impatience may easily lead to disaster.

The writer remembers encountering an unusual complication which occurred in attempting to extricate a toothplate with a coin-catcher, and it is mentioned here as proof of the statement that no one should introduce a coin-catcher unless he is prepared to proceed to further surgical measures in the event of their becoming necessary. This is what happened. A toothplate was impacted in the œsophagus at the root of the neck. It was found impossible to reach it with the ordinary œsophageal forceps, and a coin-catcher was passed. It caught in the plate, but it was then found that the plate could not be moved at all, short of using such force as would assuredly damage the œsophagus; but worse than this, it could not be disengaged from the toothplate by manipulation. It was therefore necessary to proceed to œsophagotomy at once in order to release the coin-catcher from the toothplate and remove the plate from the œsophagus.

The operation itself is comparatively simple. An incision is made parallel to the anterior border of the sterno-mastoid on the left side, because the œsophagus lies more on the left side. An opening is made which is bounded by muscles, the sterno-mastoid to the outer side, the omo-hyoid above, and the sterno-hyoid and sterno-thyroid internally. In this space the œsophagus, larynx and trachea, and the vessels are exposed. The vessels are carefully drawn to the outer side with a retractor together with the nerves.

An incision is then made into the œsophagus with scissors or scalpel right over the foreign body, which is extracted by careful manipulation. The wound in the œsophagus should be sewn up with Lembert sutures, but the external wound should be left open, as there is nearly always a good deal of leakage. In fact, some authorities are so impressed by the necessary risks involved in opening the œsophagus that they advise that, if possible, the œsophagus should only be exposed by operation, and that then by actual manipulation of its wall, an attempt should be made to free the foreign body and to extract it per os. The argument does not seem sound: the risk of making a clean incision in the œsophagus is less than that of tearing the wall by drawing up a toothplate, even if it has been freed by manipulation.

Before proceding to the extreme measure of œsophagotomy it is sometimes possible to induce the foreign body to pass into the stomach by means of pressure with a bougie; and if this manœuvre is successful, it will pass by the rectum, if it be small; or if it is too large for there to be any prospect of its doing this, it can be removed by gastrotomy. The latter operation is really preferable to œsophagotomy, because the stomach is a long-suffering organ, and is less disturbed by surgical interference than almost any other important abdominal organ.

The worst cases of all are those in which the foreign body becomes impacted in the œsophagus in the middle of its intra-thoracic course, too low to be accessible from the neck and too high to be reached from the stomach. The ultimate result of leaving the body *in situ* is a fatal termination, because it will eventually ulcerate through the œsophageal wall and set up a septic mediastinitis, or possibly make its way into the aorta. Any operative proceeding, therefore, which holds out the least prospect of success is justifiable, and thoracic œsophagotomy has been performed on several occasions. When the foreign body has been located, portions of three or four ribs are resected close to the spine, the parietal pleura is pushed out of the way, and the œsophagus is opened from behind. Unfortunately, the result of such measures is hardly less unsatisfactory than if nothing is done at all. Even if it be found feasible to remove the body, which has not always been the case, accurate adaptation of the cut edges of the œsophagus is extremely difficult, and a mediastinitis may ensue more readily than if no operation had been performed.

